# Thresher: determining the number of clusters while removing outliers

**DOI:** 10.1186/s12859-017-1998-9

**Published:** 2018-01-08

**Authors:** Min Wang, Zachary B. Abrams, Steven M. Kornblau, Kevin R. Coombes

**Affiliations:** 1 Department of Biomedical Informatics, The Ohio State University, 250 Lincoln Tower, 1800 Cannon Drive, Columbus, 43210 OH USA; 2 Mathematical Biosciences Institute, The Ohio State University, 1735 Neil Avenue, Columbus, 43210 OH USA; 3 Department of Leukemia, The University of Texas M.D. Anderson Cancer Center, 1515 Holcombe Blvd., Box 448, Houston, 77030 TX USA

**Keywords:** Clustering, Number of clusters, von Mises-Fisher mixture model, NbClust, SCOD, Gap statistics, Silhouette width

## Abstract

**Background:**

Cluster analysis is the most common unsupervised method for finding hidden groups in data. Clustering presents two main challenges: (1) finding the optimal number of clusters, and (2) removing “outliers” among the objects being clustered. Few clustering algorithms currently deal directly with the outlier problem. Furthermore, existing methods for identifying the number of clusters still have some drawbacks. Thus, there is a need for a better algorithm to tackle both challenges.

**Results:**

We present a new approach, implemented in an R package called Thresher, to cluster objects in general datasets. Thresher combines ideas from principal component analysis, outlier filtering, and von Mises-Fisher mixture models in order to select the optimal number of clusters. We performed a large Monte Carlo simulation study to compare Thresher with other methods for detecting outliers and determining the number of clusters. We found that Thresher had good sensitivity and specificity for detecting and removing outliers. We also found that Thresher is the best method for estimating the optimal number of clusters when the number of objects being clustered is smaller than the number of variables used for clustering. Finally, we applied Thresher and eleven other methods to 25 sets of breast cancer data downloaded from the Gene Expression Omnibus; only Thresher consistently estimated the number of clusters to lie in the range of 4–7 that is consistent with the literature.

**Conclusions:**

Thresher is effective at automatically detecting and removing outliers. By thus cleaning the data, it produces better estimates of the optimal number of clusters when there are more variables than objects. When we applied Thresher to a variety of breast cancer datasets, it produced estimates that were both self-consistent and consistent with the literature. We expect Thresher to be useful for studying a wide variety of biological datasets.

**Electronic supplementary material:**

The online version of this article (doi:10.1186/s12859-017-1998-9) contains supplementary material, which is available to authorized users.

## Background

Cluster analysis is the most common unsupervised learning method; it is used to find hidden patterns or groups in unlabeled data. Clustering presents two main challenges. First, one must find the optimal number of clusters. For example, in partitioning algorithms such as K-means or Partitioning Around Medoids (PAM), the number of clusters must be prespecified before applying the algorithm [1–3]. This number depends on existing knowledge of the data and on domain knowledge about what a good and appropriate clustering looks like. The mixture-model based clustering of genes or samples in bioinformatics data sets implemented in EMMIX-GENE also requires prescpecifying the number of groups [4]. Other implementations of mixture models, such as the mclust package in R [5], determine the number of clusters by using the Bayesian Information Criterion to select the best among a set of differently parameterized models. Second, the existence of “outliers” among the objects to cluster can obscure the true structure. At present, very few clustering algorithms deal directly with the outlier problem. Most of these algorithms require users to prespecify both the number *k* of clusters and the number *ℓ* (or fraction *α*) of data points that should be detected as outliers and removed. Examples of such algorithms include trimmed K-means [6], TCLUST [7], the “spurious-outliers model” [8], and k-means [9]. FLO, a refinement of k-means based on Lagrangian relaxation, can discover *k* from the data but still requires the user to specify *ℓ* [10]. The only existing method we know about that can discover both the number of clusters and the number of outliers from the data is Simultaneous Clustering and Outlier Detection (SCOD) [11]. There is a need for more and better algorithms that can tackle these two challenges in partitioning the objects.

Three popular methods to identify the correct number of clusters are (1) the elbow method, (2) the mean silhouette width [12], and (3) the gap statistic [13]. The elbow method varies the number *k* of clusters and computes the total within-cluster sum of squares (SS-within) for each *k*. One plots SS-within versus *k* and selects the location of an elbow or bend to determine the number of clusters. This method is both graphical and subjective; one disadvantage is that it relies solely on a global clustering characteristic. The silhouette method shows which objects lie well within a cluster and which are merely somewhere in between clusters. The mean silhouette width measures the overall quality of clustering; it shares the same disadvantages as the elbow method. The gap statistic compares the change in within-cluster dispersion to that expected under an appropriate null distribution. The optimal *k* should occur where the gap—the amount by which the observed value falls below the expected value—is largest. However, the gap statistics may have many local maxima of similar size, introducing potential ambiguities. Another drawback of the gap statistic is that its performance is not as good at identifying clusters when data are not well separated. In addition to these methods, many other approaches have been developed to estimate the number of clusters. A wide variety of methods are reviewed by Charrad et al. (2014) and included in an R package, NbClust [14]. However, none of these methods can detect outliers.

For biological datasets containing both samples (or patients) and features (usually genes or proteins), either the samples or the features may be the objects of interest to be clustered. Sometimes, both samples and features are clustered and displayed along with a heatmap [15]. Outliers are interpreted differently depending on what we are clustering. We view outliers among the genes or proteins as “noise” that makes no useful contribution to understanding the biological processes active in the data set. Outliers among patient samples may represent either low quality samples or “contaminated” samples, such as samples of solid tumor that are intermixed with large quantities of normal stroma. However, they may also represent rare subtypes that are present in the current data set at such low numbers that they cannot be reliably identified as a separate group.

To avoid confusion, in the rest of this paper, we will refer to the things to be clustered as *objects* and to the things used to cluster them as *variables*. Many algorithms have been developed in the context of clustering large number of objects using relatively few variables. However, there are two other important scenarios: (1) clustering patients using the expression of many genes in a typical microarray dataset, or (2) clustering a few genes or proteins, say from a single pathway, using their expression values for many patients. The performance of clustering methods that estimate the optimal number of clusters hasn’t yet been assessed extensively for these two scenarios.

In this paper, we propose a novel approach, called Thresher, that combines principal components analysis (PCA), outlier filtering, and a von Mises-Fisher mixture model. Thresher views “separating the wheat from the chaff”, where “wheat” are the good objects and “chaff” are the outliers, as essential to perform better clustering. PCA is used both for dimension reduction (which should be particularly valuable in biological applications where there are more variables than objects to cluster) and to detect outliers; a key innovation of Thresher is the idea of identifying outliers based on the strength of their contribution to PCA. In our approach, objects are first mapped to loading vectors in PC space; those that survive outlier removal are further mapped to a unit hypersphere for clustering using the mixture model. This step is also motivated by modern biological applications where correlation is viewed as the primary measure of similarity; we hypothesize that correlated objects should point in the same direction in PC space.

This article is organized as follows. Different methods to compute the number of clusters are briefly reviewed in “Methods”. In “Simulations” we perform Monte Carlo simulations to compare the performance of the Thresher algorithm to existing methods. In “Breast cancer subtypes” we apply Thresher to a wide variety of breast cancer data sets in order to estimate the number of subtypes. Finally, we conclude the paper and make several remarks in “Discussion and conclusion”. Two simple examples to illustrate the implementation and usage of the Thresher package are provided in Additional file 1.

## Methods

All simulations and computations were performed using version 3.4.0 of the R statistical software environment [16] with version 0.11.0 of the Thresher package, which we have developed, and version 3.0 of the NbClust package.

In this section, we briefly review and describe the methods that are used to estimate the number of clusters for the objects contained in a generic dataset.

### Indices of clustering validity in the NbClust package

As described in “Background”, Rousseeuw (1987) developed the mean silhouette method, and Tibshirani, Walther, and Hastie (2001) proposed the gap statistic to compute the optimal number of clusters [12, 13]. Prior to those developments, Milligan and Cooper (1985) used Monte Carlo simulations to evaluate thirty stopping rules to determine the number of clusters [17]. Thirteen of these stopping rules are implemented in either the Statistical Analysis System (SAS) cluster function or in R packages: cclust (Dimitriadou, 2014) and clusterSim (Walesiak and Dudek, 2014) [18, 19]. Furthermore, various methods based on relative criteria, which consists in the evaluation of a clustering structure by comparing it with other clustering schemes, have been proposed by Dunn (1974), Lebart, Morineau, and Piron (2000), Halkidi, Vazirgiannis, and Batistakis (2000), and Halkidi and Vazirgiannis (2001) [20–23].

Charrad and colleagues reviewed a wide variety of indices of cluster validity, including the ones mentioned above [14]. They developed an R package, NbClust, that aimed to gather all indices previously available in SAS or R packages together in a single package. They also included indices that were not implemented anywhere else in order to provide a more complete list. At present, the NbClust package includes 30 indices. More details on the definition and interpretation of the 30 indices can be found at Charrad et al. (2014) [14].

### Thresher

Here we describe the Thresher method, which consists of three main steps: principal component analysis with determination of the number of principal components (PCs), outlier filtering, and the von-Mises Fisher mixture model for computing the number of clusters.

1. **Number of Principal Components.** When clustering a small number of objects with a large number of variables, dimension reduction techniques like PCA are useful. PCA retains much of the internal structure of the data, including outliers and grouping of objects, in a way that “best” preserves the variation present in the data. Data reduction is achieved by selecting the optimal number of PCs to separate signal from noise. After standardizing the data, we compute the optimal number *D* of significant PCs using an automated adaptation of a graphical Bayesian model first described by Auer and Gervini [24]. In order to apply their model, one must decide, while looking at the graph of a step function, what constitutes a significantly large step length. We have tested multiple criteria to solve this problem. Based on a set of simulations [25], the best criteria for separating the steps into “short” and “long” subsets are:
  - **Twice Mean.** Use twice the mean of the set of step lengths as a cutoff to separate the long and short steps.
  - **Change Point (CPT).** Use the cpt.mean function from the changepoint R package to detect the first change point in the sequence of sorted step lengths.
  We have automated this process in an R package, PCDimension [25].
2. **Outlier detection.** Our method to detect outliers relies on the PCA computed in the previous step. A key point is that the principal component dimension *D* is the same for a matrix and its transpose; what changes is whether we view the objects to be clustered in terms of their projected scores or in terms of the weight they contribute to the components. Our innovation is to do the latter. In this way, each object yields a *D*-dimensional “loading” vector. The length of this vector summarizes its overall contributions to any structure present in the data. We use the lengths to separate the objects into “good” (part of the signals that we want to detect) and “bad” (the outliers that we are trying to remove). Based on simulation results that will be described in “Simulations” section, the default criterion to identify an object as an outlier is that the length is less than 0.3.
3. **Optimal number of clusters.** After removing outliers, we use the Auer-Gervini model to recalculate the number *D*_0_ of PCs for the remaining good objects, which are viewed as vectors in *D*_0_-dimensional PC space. We hypothesize that the loading vectors associated to objects that should be grouped together will point in (roughly) the same direction. So, we use the directions of the loading vectors to map the objects onto a unit hypersphere. Next, in order to cluster points on the hypersphere, we use mixtures of von Mises-Fisher distributions [26]. To fit this mixture model, we use the implementation in version 0.1-2 of the movMF package [27]. Finally, to select the optimal number of groups, we compute the Bayesian Information Criterion (BIC) for each *N* in the range *N*=*D*_0_, *D*_0_+1, …, 2*D*_0_+1; the best number corresponds to the minimum BIC. The intuition driving the restriction on the range is that we must have at least one cluster of points on the hypersphere for each PC dimension. However, weight vectors that point in opposite directions (like strongly positively and negatively correlated genes) should be regarded as separate clusters, approximately doubling the potential number of clusters. The extra +1 for the number of clusters was introduced to conveniently handle the special case when *D*_0_=0 and there is only one cluster of objects.

## Results

### Simulations

By following Monte Carlo protocols, we want to explore how well the cutoff separates signal from noise in the outlier detection step. We also study the accuracy and robustness of the different algorithms described in “Methods” section on estimating the number of clusters.

#### Selecting a cutoff via simulation

In order to find a default cutoff to separate signal from noise, we simulated five different kinds of datasets. The simulated datasets can have either one or two true underlying signals (or clusters), and each signal can either be all positively correlated or can include roughly half positive and half negative correlation. We use the following algorithm:

1. Select a number of variables for each dataset from a normal distribution with mean 300 and standard deviation 60.
2. Select an even number of objects between 10 and 20.
3. Split the set of objects roughly in half to represent two groups.
4. Independently, split the objects in half to allow for positive and negative correlation.
5. Randomly choose a correlation coefficient from a normal distribution with mean 0.5 and standard deviation 0.1.
6. For each of the five kinds of correlation structures, simulate a dataset using the selected parameters.
7. Add two noise objects (from standard normal distributions) to each data set to represent outliers.

We repeated this procedure 500 times, producing a total of 2500 simulated datasets. For each simulated dataset, each object is mapped to a loading vector in PC space; let *Δ* be its length. To separate “good” signals from “bad”, we computed the true positive and false positive rates on the ROC curve corresponding to *Δ* (Table 1). The results in this table suggest that a cutoff anywhere between 0.30 and 0.35 is reasonable, yielding a false negative rate of about 5 in 1000 and a false positive rate about 4 in 1000. We propose using the smallest of these values, 0.30, as our default cutoff, since this will eventually retain as many true positives as possible.

**Table 1:** True positive and false positive rates for *Δ* between 0.20 and 0.60

Delta	False positive rate	True positive rate
0.20	0.0300	0.9954664
0.25	0.0108	0.9954143
0.26	0.0082	0.9953882
0.27	0.0074	0.9953882
0.28	0.0068	0.9953882
0.29	0.0056	0.9953882
0.30	0.0042	0.9953882
0.31	0.0040	0.9953622
0.32	0.0038	0.9953622
0.33	0.0038	0.9953361
0.34	0.0038	0.9953361
0.35	0.0038	0.9952840
0.40	0.0036	0.9949453
0.45	0.0036	0.9939812
0.50	0.0036	0.9904898
0.55	0.0036	0.9819698
0.60	0.0036	0.9554455

#### Simulated data types

**Fig. 1:**
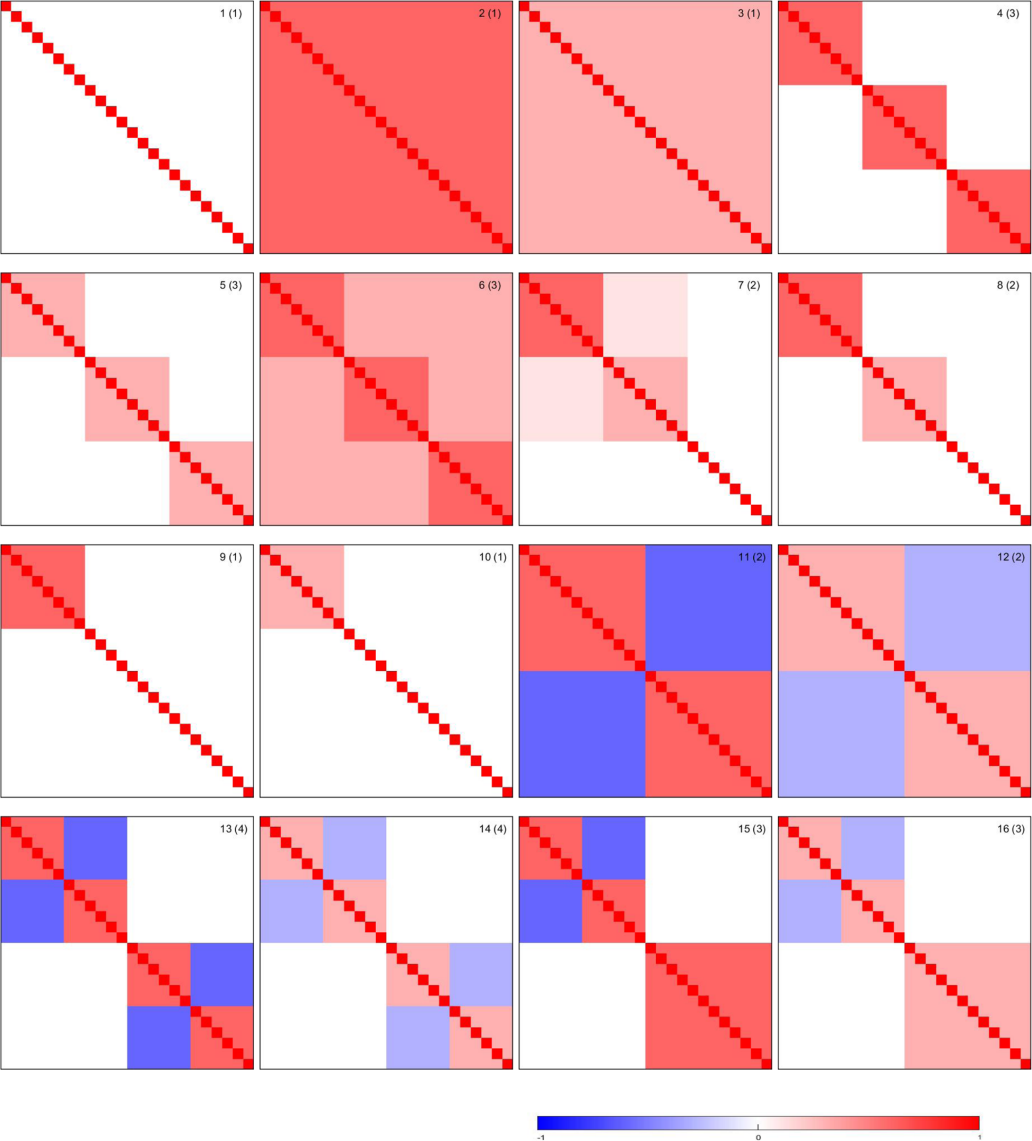
The 16 correlation matrices considered in the simulation studies. Values of correlations are provided by the colorbar. Numbers in parentheses correspond to the known numbers of clusters

Datasets are simulated from a variety of correlation structures. To explore the effects of different combinations of factors, including outliers, signed or unsigned signals, and uncorrelated variation, we use the 16 correlation matrices displayed in Fig. 1. For each correlation structure, we take the corresponding covariance matrix to be *Σ*=*σ*^2^∗corr(*X*) where *σ*^2^=1. For all 16 covariance matrices, we use the same marginal distribution–multivariate normal distribution. That is, we first randomly generate a mean vector *μ*, then sample the objects from multivariate normal distributed MVN(*μ*,*Σ*). The grouping of the objects is included in the correlation structures and those objects in different blocks are separated under Pearson distance, not necessarily under the traditional Euclidean distance. Matrix 1 contains only noise variables; it is a purely uncorrelated structure. Matrices 2 and 3 represent correlation structures with various homogeneous cross-correlation strengths (unsigned signals) 0.3 and 0.8. Matrices 4–10 are correlation matrices where between-group (0.3, 0.1, or 0) and within-group (0.8, or 0.3) correlations of variables are fixed. More details about them can be found in [28, 29]. Matrices 11–16 are correlation structures where negative cross-correlations (−0.8 or −0.3, signed signals) are considered within groups, and mixture of signed and unsigned signals are also included.

The number of objects for each simulated dataset is set to either 24 or 96. The range of 24 to 96 is chosen to represent small to moderately sized data sets. Similarly, we consider either 96 or 24 variables. A dataset with 24 variables is viewed as a small dataset; one with 96 variables, as moderate. The true number of groups (or clusters) is shown in parentheses in the plots in Fig. 1. By varying the number of objects and the number of clusters, we can investigate the effects of the number of “good” objects and the number of objects per group.

#### Empirical results and comparisons on outlier detection

The Thresher method is designed to separate “good” objects from “bad” ones; that is, it should be able to distinguish between true signal and (uncorrelated) noise in a generic dataset. To investigate its performance at identifying noise, we simulated 1000 sample datasets for each of the correlation structures 7–10 from Fig. 1. We use the definitions of sensitivity, specificity, false discovery rate (FDR) and the area under the curve (AUC) of the receiver operating characteristic (ROC) as described in Hastie et al. (2009) [30] and Lalkhen and McCluskey (2008) [31]. In particular, sensitivity is the fraction of truly “bad” objects that are called bad, and specificity is the fraction of truly “good” objects that are called good. We summarize the results for datasets 7–10 in Table 2.

**Table 2:** Summary statistics for detecting good and bad objects in datasets 7-10 from Thresher

Scenarios and datasets	96 variables, 24 objects				24 variables, 96 objects			
	Dataset 7	Dataset 8	Dataset 9	Dataset 10	Dataset 7	Dataset 8	Dataset 9	Dataset 10
Sensitivity	0.990	0.985	0.988	0.958	0.822	0.816	0.836	0.809
Specificity	0.606	0.552	1	0.999	0.688	0.655	1	0.917
FDR	0.427	0.458	0	0.001	0.399	0.426	0	0.047
AUC	0.798	0.768	0.994	0.978	0.755	0.735	0.918	0.863

Table 2 suggests that Thresher does a good job of identifying noise when there are 96 variables and 24 objects, while it performs moderately well when the datasets have 24 variables and 96 objects. The specificity statistics indicate that Thresher is able to select the true “good” objects, especially when the number of actual “good” objects is small. Furthermore, from the FDR values, we see that almost all the “noise” objects chosen by Thresher are truly “noise” in correlation structures 9 and 10, which contain a relatively large proportion of “noise” objects. For datasets 7 and 8 with a smaller fraction of “noise” objects, some “good” objects are incorrectly identified as “noise”. Their percentage is not negligible, especially when the datasets contain few variables and many objects. The AUC statistics for correlation matrices 9 and 10 are higher than those for correlation matrices 7 and 8, regardless of the relative numbers of variables and objects. That is, Thresher has higher accuracy for identifying both “good” and “bad” objects when there is a larger fraction of “bad” objects and a smaller number of clusters in the dataset. Finally, for any given correlation pattern, Thresher performs slightly better in datasets with more variables than objects, and slightly worse in datasets with fewer variables than objects.

Zemene et al. showed that their SCOD algorithm was more effective at detecting outliers than unified k-means on both real and synthetic datasets [11]. Here, we compare SCOD to Thresher on the synthetic datasets of “Simulated data types” section. The SCOD results are displayed in Table 3. By comparing Tables 2 and 3, we see that the sensitivity of Thresher is always substantially larger than that of SCOD. In other words, Thresher performs better at identifying noise than SCOD regardless of the correlation structure or the relative number of variables and objects. From the FDR values, we can tell that the proportion of true “noise” objects among those called “noise” by Thresher is higher than that from SCOD in datasets 9–10. The performance of both methods is less satisfactory for datasets 7–8 with a smaller fraction of “noise” objects. Finally, the AUC statistics from the SCOD algorithm are close to 0.5 for each correlation matrix, which suggests that Thresher produces more precise results for identifying both “good” and “bad” objects regardless of the correlation structures.

**Table 3:** Summary statistics for detecting good and bad objects in datasets 7-10 from SCOD algorithm

Scenarios and datasets	96 variables, 24 objects				24 variables, 96 objects			
	Dataset 7	Dataset 8	Dataset 9	Dataset 10	Dataset 7	Dataset 8	Dataset 9	Dataset 10
Sensitivity	0.337	0.344	0.327	0.328	0.225	0.228	0.223	0.217
Specificity	0.670	0.661	0.674	0.658	0.780	0.774	0.780	0.786
FDR	0.660	0.663	0.333	0.342	0.661	0.666	0.333	0.338
AUC	0.504	0.502	0.501	0.493	0.502	0.501	0.502	0.501

#### Number of clusters: comparing Thresher to existing methods

**Fig. 2:**
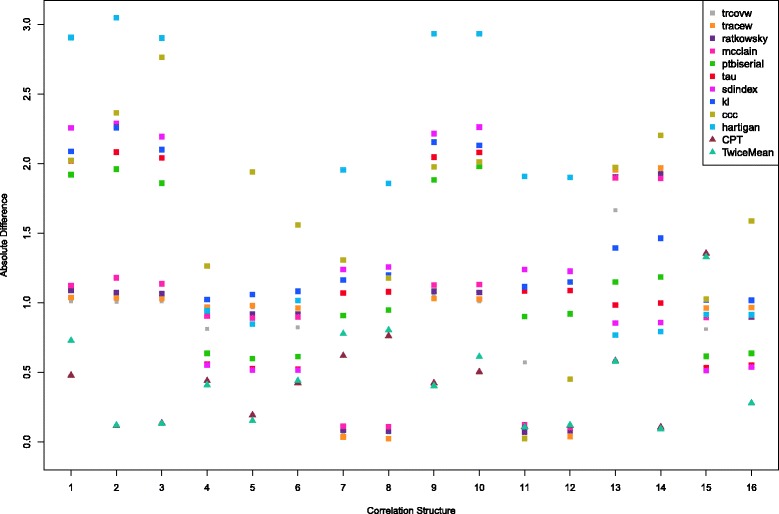
Values of the absolute difference between the estimated values and the known number of clusters across the correlation matrices for 96 variables and 24 objects

**Fig. 3:**
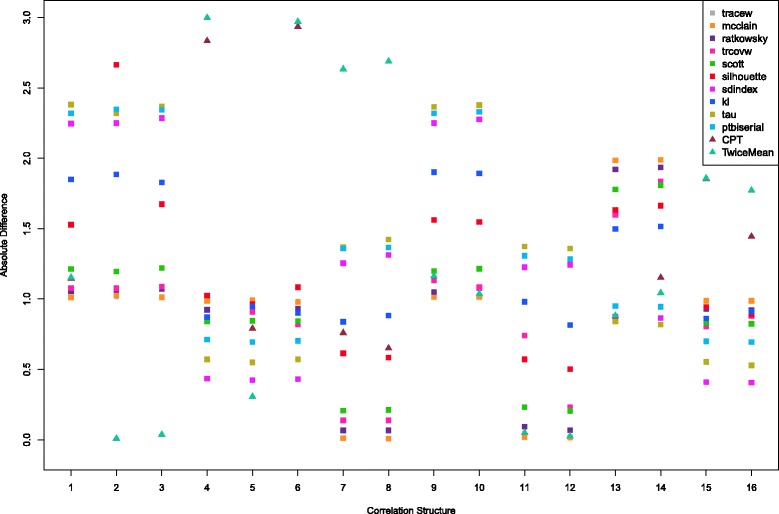
Values of the absolute difference between the estimated values and the known number of clusters across the correlation matrices for 24 variables and 96 objects

For each of the 16 correlation structures, we simulate 1000 sample datasets. Then we estimate the numbers of clusters using SCOD and all methods described in “Methods” section. For each index in the NbClust package, for two variants of Thresher, and for SCOD, we collect the estimated number of clusters for each sample dataset. We compute the average of the absolute differences between the estimated and true numbers of clusters over all 1000 simulated datasets. The results are presented in Figs. 2 and 3 and in Tables 4 and 5. For each method, we also compute the overall averages of the absolute differences (over all 16 correlation matrices) and report them in the last rows of these tables.

**Table 4:** Values of the absolute difference between the estimated and the known number of clusters across the correlation matrices for 96 variables and 24 objects

Methods	NbClust Top 10 Best Indices										Thresher		
	trcovw	tracew	ratkowsky	mcclain	ptbiserial	tau	sdindex	kl	ccc	hartigan	CPT	TwiceMean	SCOD
1	1.008	1.037	1.088	1.123	1.920	2.020	2.255	2.087	2.022	2.905	0.477	0.727	**0.150**
2	1.004	1.032	1.073	1.179	1.959	2.081	2.287	2.258	2.363	3.048	**0.114**	0.119	0.153
3	1.008	1.031	1.065	1.135	1.858	2.041	2.193	2.099	2.763	2.902	0.135	**0.130**	0.165
4	0.811	0.968	0.921	0.904	0.635	0.559	0.551	1.023	1.263	0.941	0.438	**0.408**	1.887
5	0.965	0.978	0.918	0.888	0.598	0.524	0.516	1.058	1.940	0.846	0.192	**0.150**	1.882
6	0.822	0.960	0.917	0.897	0.613	0.522	0.516	1.082	1.558	1.016	**0.423**	0.438	1.897
7	0.064	**0.034**	0.082	0.112	0.906	1.068	1.239	1.163	1.307	1.954	0.618	0.776	0.890
8	0.068	**0.021**	0.075	0.108	0.946	1.078	1.255	1.199	1.177	1.857	0.760	0.802	0.914
9	1.05	1.029	1.082	1.126	1.882	2.045	2.215	2.153	1.975	2.933	0.422	0.401	**0.163**
10	1.011	1.025	1.072	1.130	1.981	2.080	2.262	2.129	2.011	2.932	0.502	0.611	**0.179**
11	0.571	0.024	0.069	0.123	0.900	1.084	1.239	1.114	**0.021**	1.906	0.109	0.104	0.918
12	0.104	**0.038**	0.081	0.101	0.919	1.088	1.225	1.148	0.450	1.901	0.115	0.120	0.913
13	1.664	1.956	1.902	1.897	1.147	0.983	0.852	1.392	1.971	0.767	0.582	**0.576**	2.930
14	1.938	1.969	1.925	1.893	1.184	0.997	0.857	1.463	2.201	0.793	0.105	**0.092**	2.884
15	0.810	0.960	0.902	0.892	0.614	0.531	**0.512**	1.020	1.025	0.914	1.354	1.328	1.910
16	0.958	0.964	0.896	0.906	0.635	0.549	0.537	1.017	1.586	0.912	**0.277**	0.278	1.897
Average	0.866	0.877	0.879	0.901	1.169	1.203	1.282	1.463	1.602	1.783	**0.414**	0.441	1.233

Bold values indicate the best results for row settings

**Table 5:** Values of the absolute difference between the estimated and the known number of clusters across the correlation matrices for 24 variables and 96 objects

Methods	NbClust Top 10 Best Indices										Thresher		
	tracew	mcclain	ratkowsky	trcovw	scott	silhouette	sdindex	kl	tau	ptbiserial	CPT	TwiceMean	SCOD
1	**1.008**	1.011	1.055	1.078	1.213	1.528	2.246	1.850	2.382	2.320	1.147	1.153	1.011
2	1.011	1.024	1.064	1.076	1.195	2.665	2.250	1.885	2.320	2.348	**0.009**	**0.009**	1.021
3	1.018	1.013	1.070	1.088	1.221	1.674	2.285	1.829	2.369	2.344	**0.037**	**0.037**	0.988
4	0.987	0.988	0.923	0.849	0.840	1.024	**0.435**	0.871	0.571	0.711	2.834	2.999	1.013
5	0.982	0.994	0.936	0.908	0.845	0.962	0.424	0.947	0.550	0.695	0.790	**0.307**	1.008
6	0.989	0.979	0.933	0.820	0.844	1.083	**0.432**	0.901	0.572	0.703	2.935	2.971	1.011
7	**0.011**	0.012	0.067	0.137	0.206	0.614	1.254	0.838	1.369	1.360	0.760	2.634	0.318
8	0.015	**0.008**	0.067	0.138	0.212	0.585	1.314	0.882	1.422	1.366	0.650	2.689	0.346
9	1.014	1.016	1.050	1.133	1.198	1.562	2.250	1.902	2.365	2.320	1.163	1.163	**0.981**
10	1.011	1.015	1.078	1.083	1.215	1.547	2.277	1.893	2.378	2.330	1.035	1.037	**1.009**
11	0.023	**0.020**	0.094	0.741	0.231	0.571	1.226	0.981	1.373	1.308	0.050	0.049	0.350
12	**0.016**	0.017	0.068	0.232	0.205	0.503	1.244	0.816	1.359	1.285	0.025	0.025	0.372
13	1.983	1.985	1.920	1.597	1.779	1.634	**0.875**	1.497	0.839	0.951	0.878	0.881	2.006
14	1.987	1.988	1.936	1.834	1.809	1.663	**0.866**	1.515	0.819	0.945	1.152	1.043	2.006
15	0.983	0.988	0.928	0.805	0.831	0.942	**0.410**	0.861	0.554	0.699	1.853	1.858	1.033
16	0.987	0.987	0.922	0.878	0.824	0.892	**0.406**	0.910	0.529	0.693	1.444	1.772	1.005
Average	**0.877**	0.878	0.882	0.900	0.917	1.216	1.262	1.274	1.361	1.399	1.048	1.289	0.967

Bold values indicate the best results for row settings

In Fig. 2 (and Table 4), we consider the scenario when the datasets contain 96 variables and 24 objects. We display the results for both Thresher variants and for the 10 best-performing indices in NbClust. In Fig. 3 (and Table 5), there are 24 variables and 96 objects for all the datasets. The results in the tables can help determine how well each method performs among all correlation structures and whether the proposed Thresher method is better than the indices in NbClust package on computing the number of clusters. The closer to zero the value in the tables is, the better the method will be for the corresponding correlation structure.

From Fig. 2 and Table 4, we see that Thresher, using either the CPT or the TwiceMean criterion, performs much better than the best 10 indices in the NbClust package across the correlation structures. It produces the most accurate estimates on average over the 16 possible correlation structures. In each row of the table, the smallest value, corresponding to the best method, is marked in bold. For 8 of the 16 correlation structures, one of the Thresher variants has the best performance. For correlation structures 7, 8, 11 and 12, either the TraceW index or the Cubic Clustering Criterion (CCC) index performs best. Even though the Trcovw index is not the best performer for any of the individual correlation structures, it produces the most accurate overall results among all 30 indices in the NbClust package.

Figure 3 and Table 5 suggest that Thresher, with either the CPT or TwiceMean criterion, performs slightly worse than the best 5 indices—Tracew, McClain, Ratkowsky, Trcovw and Scott—in the package NbClust, when averaged over all correlation structures with 24 variables and 96 objects. The Tracew index produces the best result on average; the overall performance of the McClain and Ratkowsky indices is similar to that of the Tracew index. As before, the smallest value corresponding to the best method in each row of the table is marked in bold. As we can see, either the Tracew or the McClain index performs the best for the correlation structures 1, 7, 8, 11 and 12. For datasets with correlation structures 4, 6 and 13–16, the Sindex index yields the most accurate estimates. However, for correlation structures 2, 3 and 5, one of the Thresher variants performs best. Even though Thresher performs slightly worse than the five best indices, it still outperforms the majority of the 30 indices in the NbClust package.

Moreover, the number of clusters computed by Thresher and SCOD for each scenario and dataset are provided and compared in Tables 4 and 5. From Table 4, one can see that Thresher gives us much more accurate estimates than SCOD does on average over all 16 correlation structures with 24 objects and 96 variables. More specifically, Thresher performs better than SCOD for all possible datasets except those with correlation structures 1, 9 and 10. For datasets with 96 objects and 24 variables as showed in Table 5, Thresher is slightly worse than SCOD in estimating the number of clusters when averaging over all 16 correlation structures. However, Thresher yields more precise estimates than SCOD does for all datasets except those with correlation structures 1, 4, 6–10, 15 and 16.

#### Running time

In addition to the comparisons of outlier detection and determination of number of clusters, we computed the average running time of the methods including the NbClust indices with top performance over all correlation matrices per data set (Table 6). All timings were carried out on a computer with an Intel®; Xeon®; CPU E5-2603 v2 @ 1.80 GHz processor running Windows®; 7.1. The table suggests that the computation time increases as the number of objects increases for Thresher, SCOD, and NbClust indices McClain, Ptbiserial, Tau, and Silhouette. From the table, we can see that SCOD uses the least time in computing the number of clusters when there are 24 objects and 96 variables in the dataset. For datasets with 96 objects and 24 variables, NbClust indices Trcovw, Tracew, CCC and Scott spend the least time. Thresher takes more time than most of the other algorithms tested, which is likely due to fitting multiple mixture models to select the optimal number of clusters.

**Table 6:** Average running time of the methods (Thresher, SCOD and the indices in NbClust with top performance) across correlation matrices (unit: seconds)

Rules	NbClust							
	trcovw	tracew	ratkowsky	mcclain	ptbiserial	tau	sdindex	kl
96 var., 24 obj.	0.09	0.09	0.116	0.029	0.029	0.077	0.291	0.275
24 var., 96 obj.	0.025	0.025	0.055	0.039	0.165	1.780	0.113	0.109
Rules	NbClust				Thresher		SCOD	
	ccc	hartigan	scott	silhouette	CPT	TwiceMean	SCOD	
96 var., 24 obj.	0.088	0.169	0.092	0.027	0.25	0.271	0.009	
24 var., 96 obj.	0.025	0.075	0.025	0.071	0.419	0.530	0.057	

### Breast cancer subtypes

One of the earliest and most significant accomplishments when applying clustering methods to transcriptomics datasets was the effort, led by Chuck Perou, to understand the biological subtypes of breast cancer. In a series of papers, his lab used the notion of an “intrinsic gene set” to uncover at least four to six subtypes [32–35]. We decided to test whether Thresher or some other method can most reliably and reproducibly find these subtypes in multiple breast cancer datasets. All datasets were downloaded from the Gene Expression Omnibus (GEO; http://www.ncbi.nlm.nih.gov/geo/). We searched for datasets that contained the keyword phrases “breast cancer” and “subtypes”, that were classified as “expression profiling by array” on humans, and that contained between 50 and 300 samples. We then manually removed a dataset if the study was focused on specific subtypes of breast cancer, as this would not represent a typical distribution of the full cohort of breast cancer samples. After this step, we were left with 25 datasets. The primary microarray data are available in GEO under the following accession numbers: GSE1992, GSE2607, GSE2741, GSE3143, GSE4611, GSE10810, GSE10885, GSE12622, GSE19177, GSE19783, GSE20711, GSE21921, GSE22093, GSE29431, GSE37145, GSE39004, GSE40115, GSE43358, GSE45255, GSE45827, GSE46184, GSE50939, GSE53031, GSE56493, GSE60785.

To select the genes that best characterize the tumor subtypes, we rely on the intrinsic analysis performed by Sorlie et al. (2003) and Hu et al. (2006) [33, 34]. They compared “within class” to “across class” variation to identify genes that show low variability within replicates, but high variability across different tumors. Using a 105-tumor training set containing 26 replicate sample pairs, they derived an initial breast tumor gene list (the Intrinsic/UNC (The University of North Carolina at Chapel Hill) list) that contained 1300 genes [34]. They tested this list as a predictor of survival on tumors from three independent microarray studies, and focused on a subset of genes. In this way, they produced a new “intrinsic gene list” containing 306 genes that they used to perform hierarchical clustering. This new intrinsic gene list had an overlap of 108 genes with a previous breast tumor gene set (the Intrinsic/Stanford list) from Sorlie et al. (2003) [33]. They also showed that this new intrinsic gene list reflects the “intrinsic” and stable biological properties of breast tumors. It typically identifies distinct subtypes that have prognostic significance, even though no knowledge of outcome was used to derive this gene set [33, 34].

**Fig. 4:**
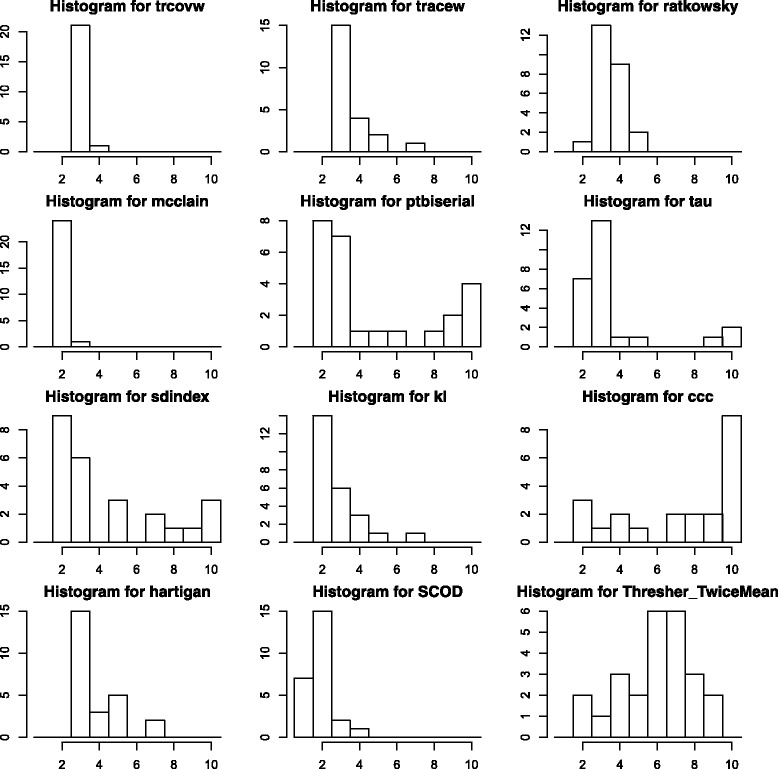
Comparison of top NbClust indices with Thresher (TwiceMean) and SCOD on estimating the number of clusters from GEO breast cancer datasets

We used the new Intrinsic/UNC list to cluster samples in each of the 25 breast cancer data sets from GEO. In these datasets, the number of genes (variables) is always greater than the number of samples (objects). For this analysis, we used the 10 best indices from the NbClust package (corresponding to Fig. 2). The performance of these indices is compared to Thresher and SCOD for computing the number of clusters in the GEO datasets. We plot a histogram of the predicted cluster numbers across the 25 datasets for each method in Fig. 4. The results including the number of clusters and outliers for analyzing the breast cancer data sets via Thresher are also provided in Table 7.

**Table 7:** Summary of the data and analysis in clustering breast cancer subtypes

Dataset	Sample #	Outlier #	Outlier percentage	Cluster #
GSE60785	55	10	18.18	2
GSE43358	57	5	8.77	3
GSE10810	58	0	0.00	6
GSE29431	66	2	3.03	9
GSE50939	71	1	1.41	2
GSE39004	72	9	12.50	4
GSE46184	74	8	10.81	4
GSE19177	75	2	2.67	4
GSE37145	76	0	0.00	7
GSE21921	85	3	3.53	6
GSE20711	90	18	20.00	7
GSE40115	92	1	1.09	5
GSE12622	103	0	0.00	7
GSE22093	103	3	2.91	5
GSE19783	115	1	0.87	8
GSE56493	120	6	5.00	6
GSE10885	125	4	3.20	7
GSE2607	126	3	2.38	7
GSE45255	139	10	7.19	8
GSE45827	155	9	5.81	6
GSE3143	158	6	3.80	7
GSE53031	167	10	5.99	6
GSE2741	169	7	4.14	6
GSE1992	170	8	4.71	8
GSE4611	218	23	10.55	9

Based on the literature [32, 34–40], we believe that a reasonable and coherent estimate for the number of clusters in a representative sample of breast tumors ranges from 4 to 7. From the histograms in Fig. 4, one can see that the indices in the NbClust package either underestimate or overestimate the number of clusters to some extent. SCOD is very conservative; it tends to consistently underestimate the number of clusters. By contrast, the Thresher method with criterion “TwiceMean” produces more robust, consistent, and accurate estimates for the number of clusters in these datasets. Thresher is the only method that produces estimates on the number of clusters centered in the “consensus” range from 4 to 7.

## Discussion and conclusion

In this paper, in order to solve both of the main challenges in clustering—selecting the optimal number of clusters and removing outlier objects—we propose a novel method called “Thresher”. For a generic dataset, Thresher can help select and filter out noise, estimate the optimal number of clusters for the remaining good objects, and finally perform grouping based on the von Mises-Fisher mixture model.

Unlike most other clustering methods, Thresher can reliably detect whether the objects of interest are “good” or “bad” (outliers). The results of our computational experiment (shown in Table 2) for datasets with correlation structures 7–10 show that Thresher does an excellent job at detecting outliers. In a head-to-head comparison with SCOD, the only previously published algorithm that can simultaneously determine the number of clusters and the number of outliers, Thresher was consistently better at outlier detection by every measure. (Compare the Thresher results above to the SCOD results in Table 3.)

We started this project by hypothesizing that removing outliers would improve the ability of Thresher to accurately estimate the number of clusters. To test that ability, we compared the performance of Thresher both to SCOD and to all 30 indices implemented in the NbClust package. Toward this end, we simulated datasets of different “shapes” (defined by the relative number of objects and variables) and different correlation structures. Critically, we found that changes in both the shape and the correlation structure lead to large differences in performance. Thresher is clearly best when there are more variables than objects to cluster. Its performance is solid (in the top six or seven of the 30 + algorithms tested) but not exceptional when there are more objects than variables.

Historically, most clustering algorithms have been developed and tested in the situation when there are more objects than variables. Many of the classic examples to test clustering algorithms simulate a large number of objects in only two or three dimensions. By contrast, modern applications of clustering in the “omics” settings common to molecular biology work in a context with many more variables than objects. In these kinds of settings, the “curse of dimensionality” suggests that objects are likely to be so scattered that everything looks like an outlier. Some of the key aspects of the Thresher method—the use of principal components for dimension reduction and the focus on identifying true outliers—were motivated by our desire to apply clustering in omics settings. Our findings show that Thresher will work better than the existing algoirthms in this context. They also suggest, however, that an opportunity still exists to develop and optimize better clustering algorithms for this challenging setting.

Correlation stuctures also have a significant impact on the perfomance of clustering algorithms. When clustering a dataset with fewer objects than variables, Thresher is either the best or second best method for correlation structures 1–6, 9–10, 13–14, and 16. (SCOD, the other method that detects and removes outliers, is best for correlations 1, 9, and 10.) These correlation structures are characterized by the presence of blocks of correlated objects, or a relatively large proportion of outliers, or more than one mixture of signed and unsigned signals. The indices Tracew, Trcovw, Ratkowsky and McClain produce better estimates for correlation structures 7–8 where there is a relative small proportion of outliers. For correlation structures 11–12 with only one mixture of signed and unsigned signals, Tracew, Ratkowsky, McClain and Thresher do well. Sdindex only gives us the best estimates for the number of clusters in datasets of correlation structure 15.

When there are more objects than variables, the Tracew index in the NbClust package produces the best estimates on average. Looking at the various correlation structures, we find that Tracew, McClain, and Ratkowsky perform well for correlation structures 1 and 7–12. In other words, they produce highly accurate results in estimating the number of clusters when the objects include some outliers or there is exactly one strong cluster containing a mixture of signed and unsigned signals. Thresher is the best for datasets of correlation structures 2, 3 and 5 whose objects have one big block or several uncorrelated blocks of weak within-group correlation. SCOD again has the best performance for correlation structures 9 and 10. The indices Sdindex and Tau perform best for correlation structures 4, 6 and 13–16 where there are several blocks of objects with high within-group correlation or more than one mixture of signed and unsigned signals.

The fact that correlation structures have a strong effect on the performance of clustering algorithms presents a challenge for users wanting to apply these algorithms. When we sit down to cluster a dataset, we already know if we have more objects or more variables, so we can choose our methods accordingly. But we do not know the true underlying correlation structure, and so we cannot use that information to guide our choice of algorithm. In this manuscript, we have dealt with that issue by computing the average performance over a range of different correlation structures. Based on those averages, we recommend using Thresher as the method of choice for determining the number of clusters whenever there are more variables than objects. When there are more objects than variables, Thresher still outperforms the majority of the 30 indices in the NbClust package. In this case, we expect Thresher to give reasonable answers, but with a reasonable chance that it will be off by about one.

We also applied Thresher to 25 breast cancer datasets downloaded from GEO in order to investigate the consistency and robustness of the number of clusters defined by the intrinsic gene list. To our knowledge, this is the most comprehensive study of the breast cancer subtypes defined by a single gene list across multiple data sets. The consensus answer in the literature for the “optimal” number of breast tumor subtypes ranges from 4 to 7. When applied to the GEO datasets, the best indices from the NbClust package either underestimate or overestimate the number of subtypes. Some of these methods always err in the same direction; others switch between overestimating in some datasets to underestimating in others. And SCOD always underestimates the number of clusters. Only the Thresher method produces estimates that are centered in the expected range of values. This analysis suggests that Thresher performs much better than the methods from NbClust when computing the optimal number of groups in real data derived from gene expression profiling experiments.

## Additional files

Additional file 1 Using the Thresher Package. This file is porovided as a PDF file illustrating the use of the Thresher package with soime simple examples. (PDF 153 kb)

Additional file 2 R Code for Analyses. This is a zip file containing all of the R code used to perform simulations and to analyze the breast cancer data. (ZIP 407 kb)

## Abbreviations

AUC: Area under the curve
BIC: Bayesian information criterion
CCC: Cubic clustering criterion
CPT: Change point
FDR: False discovery rate
GEO: Gene expression Omnibus
PAM: Partition around Medoids
PC: Principal component
PCA: Principal components analysis
ROC: Receiver operating characteristic
SAS: Statistical Analysis System
SS-within: Within-cluster sum of squares
UNC: The University of North Carolina at Chapel Hill
