# Supplementary material for: Thresher: determining the number of clusters while removing outliers
Source: BMC Bioinformatics. 2018 Jan 8;19:9. doi: 10.1186/s12859-017-1998-9 (PMC5759208; doi:10.1186/s12859-017-1998-9)
Supplement: Supplementary file 1 — Using the Thresher Package. This file is porovided as a PDF file illustrating the use of the Thresher package with soime simple examples. (PDF 153 kb) [file 12859_2017_1998_MOESM1_ESM.pdf]

Using the Thresher Package

M Wang, ZB Abrams, SM Kornblau, and KR Coombes

December 10, 2017

1 Implementation in Thresher

In this section, we describe how to calculate the number of clusters using the R package `Thresher`, which is available from the R-Forge webpage (`http://r-forge.r-project.org/R/?group_id=1900`). We illustrate this method by exploring two small simulated datasets: an unstructured dataset that only contains random noise objects, and a structured dataset with two groups of good objects. First of all, we load all R library packages that are needed for this analysis. Note that `Thresher` implements our proposed clustering approach, while `NbClust` (developed by Charrad et al.) is used to run 30 existing indices including the gap statistics and the mean silhouette method.

1.1 Unstructured Dataset Example

1.1.1 Thresher Method

First we load the R library packages `Thresher`, `MASS` and `NbClust` in the illustration examples.

```
library(Thresher)
library(NbClust)
```

Then we simulate an unstructured dataset with 12 noise objects and 100 samples. That is, there is no pattern of groups or clusters for all the objects in the data.

```
set.seed(3928270)
ranData <- matrix(rnorm(100*12), ncol=12)
colnames(ranData) <- paste("G", 1:12, sep='')
```

Next we apply the “Thresher” function to the unstructured data and obtain the results of clustering in the saved “Thresher” and “Reaper” objects.

```r
thresh1 <- Thresher(ranData)
reap1 <- Reaper(thresh1)
```

Notice that a filtering procedure is executed in the package, and the noise objects that have contributed variation proportion less than the default threshold are removed. The noise objects are

```r
colnames(ranData)[!reap1@keep]

## [1] "G2"  "G3"  "G4"  "G6"  "G7"  "G8"  "G9"  "G12"
```

The remaining 4 objects are treated as “good” ones, and their projections onto the Bayesian principal components (PCs) space are plotted in Figure 5.

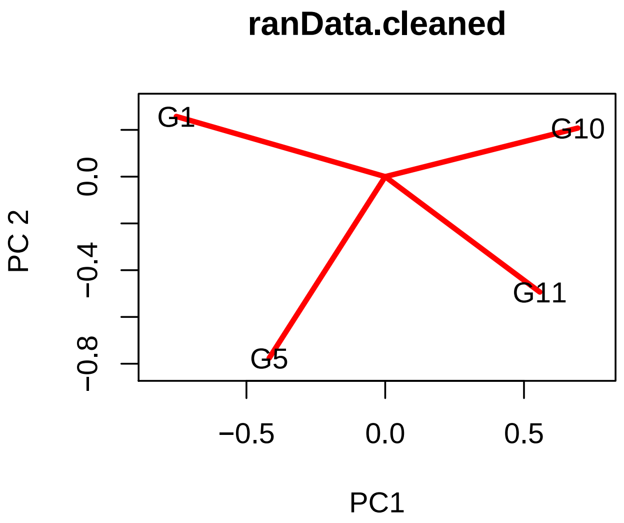

Figure 1: Plot of Objects on Projected PC Space in Unstructured Dataset

The optimal number of groups for the remaining 4 objects is estimated to be

```r
reap1@nGroups

## [1] 1
```

Finally we use the heatmap function on the “Reaper” object to look at the heatmap of the unstructured dataset with only good objects in Figure 6.

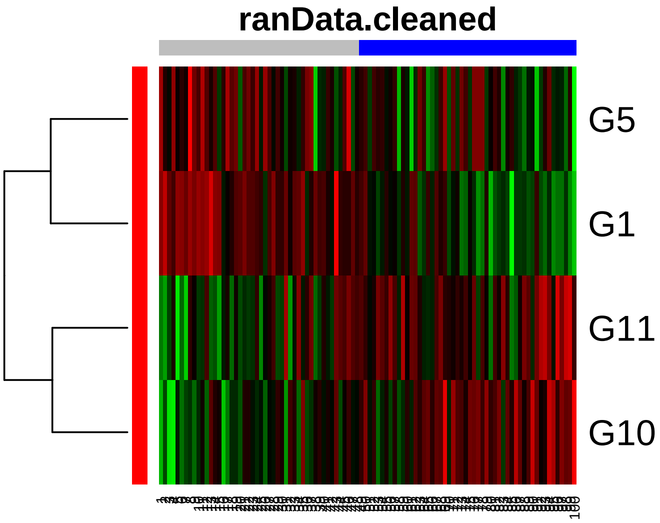

Figure 2: Heatmap of Unstructured Dataset

To sum up, in this simulation, there are 12 objects which are all noise in the unstructured dataset. The Thresher method successfully identified 8 out of 12 noise objects. For the remaining 4 "good" objects, the estimated optimal number of clusters is 1 which is reasonable, since the 4 objects are isotropically distributed which naturally form a large cluster.

1.1.2 NbClust Indices

Notice that not all indices in the `NbClust` package work for this simulated dataset. We apply the usable Tracew index in `NbClust` package to the unstructured dataset, and obtain the estimated number of clusters to be 2, which is a little different from the true number 1 of clusters.

```
nbclust1 <- NbClust(t(ranData), distance="euclidean", min.nc=1,
         max.nc=10, method="ward.D2", index="trcovw")
nbclust1$Best.nc

## Number_clusters     Value_Index
##          2.0000        214.3595
```

1.2 Example Dataset with Specified Structure

1.2.1 Thresher Method

We use another example dataset with special correlation structures. In this dataset, 16 objects are generated and they are grouped into 2 clusters. Within each cluster, the objects are moderately correlated with strength $\rho = 0.5$. The between-cluster correlation is set to be 0; that is, the two clusters of objects are uncorrelated with each other.

```
set.seed(3757871)
rho <- 0.5
nProtein <- 16
splinter <- sample((nProtein/2) + (-3:3), 1)
sigma1 <- matrix(rho, ncol=nProtein, nrow=nProtein)
diag(sigma1) <- 1
sigma2 <- sigma1
sigma2[(1+splinter):nProtein, 1:splinter] <- 0
sigma2[1:splinter, (1+splinter):nProtein] <- 0
```

The "SimThresher" function combines the example dataset generation and first step of Thresher method. We again applied the Thresher method to this structured data and obtain the results of clustering in the following "Thresher" and "Reaper" objects. The true clustering of the objects is also displayed.

```
thresh2 <- SimThresher(sigma2, nSample=300)
summary(thresh2@delta)

##    Min. 1st Qu.  Median    Mean 3rd Qu.    Max.
##  0.6881  0.7472  0.7606  0.7535  0.7663  0.7782

reap2 <- Reaper(thresh2)
colnames(reap2@data)[1:splinter]

## [1] "Pr1" "Pr2" "Pr3" "Pr4" "Pr5" "Pr6" "Pr7"

colnames(reap2@data)[(splinter+1):nProtein]

## [1] "Pr8"  "Pr9"  "Pr10" "Pr11" "Pr12" "Pr13" "Pr14" "Pr15" "Pr16"
```

There are no noise objects in the simulated dataset, and this is correctly inferred by the Thresher method.

```
colnames(reap2@data)[!reap2@keep]

## character(0)
```

The number of PCs is estimated to be 2 which is the same as the known number of groups in the structured dataset.

```
reap2@pcdim

## [1] 2
```

All objects in the structured data are "good". Their projections onto the principal components (PCs) space are provided in Figure 7.

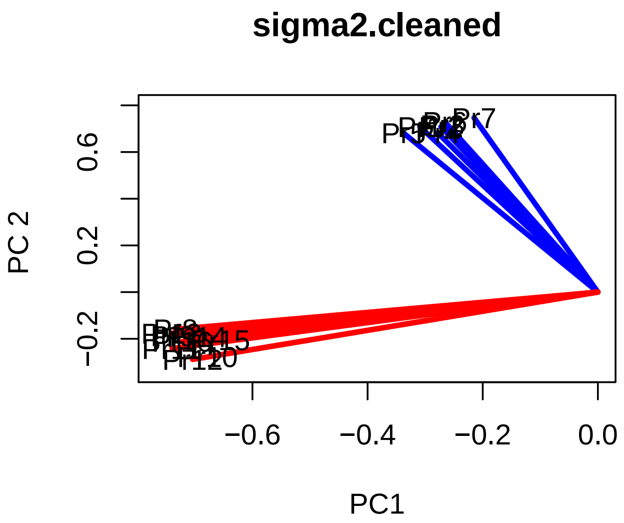

Figure 3: Plot of Objects on Projected PC Space in Structured Dataset

And the number of groups is estimated to be

```
reap2@nGroups

## [1] 2
```

Finally we apply the heatmap function to the "Reaper" object to obtain the heatmap of the structured dataset with all good objects in Figure 8. And it's clear to see that the clustering of

the objects is performing fairly well. That is, all objects are correctly grouped together into the clusters where they are supposed to be.

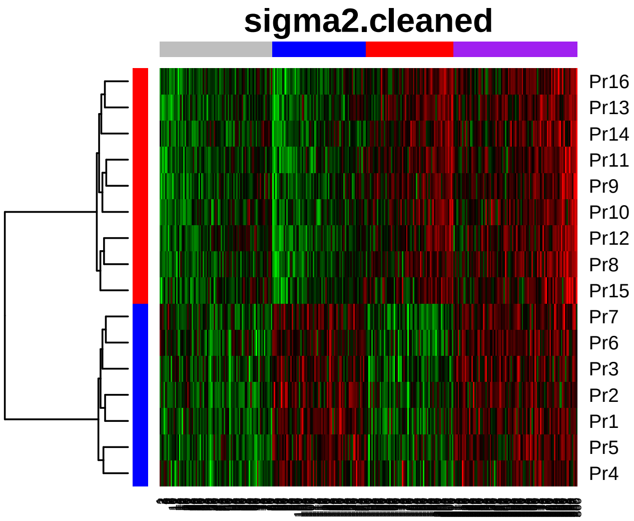

Figure 4: Heatmap of Structured Dataset

To conclude, for this simulated dataset with special structure, the Thresher method correctly identify the "good" objects and the optimal number of groups for the objects, which suggests that the Thresher method is viable and useful.

1.2.2 NbClust Indices

We apply the Tracew index in package `NbClust` to the structured dataset and obtain the estimated number of clusters to be 2 which is the same as the known number of clusters.

```
nbclust2 <- NbClust(t(thresh2@data), distance="euclidean", min.nc=1,
         max.nc=10, method="ward.D2", index="tracew")
nbclust2$Best.nc

## Number_clusters     Value_Index
##           2.000        1087.593
```
